# Divergent Intralesional Enhancement Kinetics in a Primary Ovarian Leiomyoma on Dual-Layer Spectral CT: A Case Report

**DOI:** 10.3390/diagnostics16203420

**Published:** 2026-10-21

**Authors:** Luis Miguel Granja Villavicencio, Andrea Estefanía Zurita Beltran, Alex Bernardo Hugo Merino

**Affiliations:** 1 Radiology Department, Instituto Oncológico Nacional Dr. Juan Tanca Marengo, Sociedad de Lucha Contra el Cáncer del Ecuador (SOLCA), Guayaquil 090505, Ecuador; 2 Diagnostic Imaging Specialty Program, School of Postgraduate Health Studies, Universidad de Especialidades Espíritu Santo (UEES), Samborondón 092301, Ecuador; andreazurita@uees.edu.ec; 3 Postgraduate Faculty, Radiology Program, Universidad de Las Américas (UDLA), Quito 170124, Ecuador; alex.hugo@udla.edu.ec

**Keywords:** spectral computed tomography, dual-layer CT, ovarian leiomyoma, iodine concentration, effective atomic number, electron density, intralesional heterogeneity, adnexal mass

## Abstract

**Background and Clinical Significance:** Indeterminate solid adnexal masses can remain difficult to characterize on conventional CT. Dual-layer spectral CT provides iodine concentration (IC), effective atomic number (Zeff), and electron density relative to water (EDW) from the same acquisition. We report a histopathologically confirmed primary ovarian leiomyoma and describe its regional multiphasic spectral behavior. **Case Presentation:** A 37-year-old woman presented with an O-RADS 4 solid right adnexal mass. Retrospective analysis sampled two enhancement-defined intralesional compartments across true noncontrast (TNC), arterial, venous, and delayed phases. On TNC images, ROI-A and ROI-B showed closely similar Zeff (7.39 vs. 7.37) and EDW (103.8% vs. 103.9%). ROI-A showed intense arterial enhancement (102.2 HU; IC 2.95 mg/mL; Zeff 8.78) followed by decline, whereas ROI-B showed low early enhancement and marked delayed fill-in, with delayed values slightly exceeding ROI-A (83.8 vs. 71.3 HU; IC 1.74 vs. 1.54 mg/mL; Zeff 8.25 vs. 8.16). Initial histopathology suggested fibroma; immunohistochemistry established ovarian leiomyoma. **Conclusions:** The two enhancement-defined compartments showed closely similar baseline spectral measurements but divergent postcontrast behavior. EDW demonstrated substantially lower-amplitude phase-related changes than IC and Zeff. These findings are descriptive and do not establish a diagnostic spectral signature or histologic specificity. When multiphasic dual-layer spectral CT has already been clinically acquired, regional spectral analysis may provide quantitative characterization of intralesional enhancement heterogeneity. In this case, however, spectral CT did not establish the diagnosis or alter management, and definitive diagnosis remained dependent on histopathology and immunohistochemistry.

## 1. Introduction

Solid adnexal masses remain a diagnostic challenge in cross-sectional imaging. Conventional CT characterizes tissue primarily through a single attenuation value, which limits tissue-specific differentiation [1,2]. In adnexal masses, benign and malignant lesions frequently show overlapping attenuation, and characterization relies largely on morphology [3,4]. Spectral CT parameters have not yet been incorporated into clinical guidelines, although preliminary ovarian-tumor studies have reported differences in iodine concentration and effective atomic number between benign and malignant lesions [5], and differences in iodine concentration between borderline and malignant epithelial tumors [6]. Ovarian fibroma and primary ovarian leiomyoma are benign stromal tumors with overlapping imaging features and are definitively distinguished by histopathology and immunohistochemistry [4,7].

Dual-layer spectral CT simultaneously acquires high- and low-energy photon data, enabling retrospective generation of iodine-density, Zeff, EDW, and virtual monoenergetic results [8,9]. The quantitative performance of these parameters has been evaluated in phantom studies [10,11,12,13,14], and Zeff and electron density have been investigated for tissue characterization in other organ systems [15,16,17,18,19,20]. To our knowledge, no previous report has described paired multiphasic IC, Zeff, and EDW measurements in a histologically confirmed primary ovarian leiomyoma. We report a case in which the clinical CT interpretation favored a benign stromal tumor, followed by a retrospective three-slice paired-compartment analysis of an already acquired multiphasic dual-layer spectral CT examination to characterize regional enhancement kinetics and phase-dependent spectral behavior.

## 2. Case Presentation

### 2.1. Clinical Presentation

A 37-year-old woman presented with cyclical pelvic pain. Laboratory evaluation showed an elevated CA 72-4 level (45.64 U/mL), with normal CA-125 and HE4 levels. CA 72-4 is a nonspecific tumor marker that may be elevated in benign gynecologic and gastrointestinal conditions as well as in malignancy; therefore, the isolated elevation was not considered diagnostic. Transvaginal ultrasound demonstrated a solid hypoechoic adnexal mass measuring 50 × 23 mm, with posterior acoustic shadowing and moderate internal vascularity (color score 3), classified as O-RADS 4 [21]. The patient subsequently underwent a multiphasic dual-layer spectral CT examination, which demonstrated a right adnexal lesion measuring approximately 6.0 × 3.0 × 3.8 cm. MRI was recommended but was not performed because the patient elected direct surgical management to exclude malignancy.

### 2.2. Imaging Acquisition and Spectral Analysis

Imaging was performed on a dual-layer spectral CT system (Spectral CT 7500, Philips Healthcare, Best, The Netherlands) using the institutional standard multiphasic abdominopelvic protocol, comprising TNC, arterial, venous, and delayed postcontrast phases. All phases were acquired at 120 kV, with a tube current-time product of 214, 216, 214, and 217 mAs for the TNC, arterial, venous, and delayed phases, respectively. The contrast-related phases had a CTDIvol of 16.8 mGy per phase. Ioversol (Optiray 320, 320 mgI/mL) was administered intravenously using a power injector as a single-phase injection of approximately 80 mL at 2.5 mL/s; the injector was manually initiated by the technologist. Postcontrast acquisitions were also manually triggered according to the institutional protocol. The nominal delays from the start of contrast injection were approximately 35 s for the arterial phase, 70 s for the venous phase, and 5–6 min for the delayed phase. Exact patient-specific injection-to-acquisition delays could not be verified because a contrast-bolus start timestamp was not stored in the available DICOM metadata; therefore, these values represent protocol-level timing. Patient-specific DICOM acquisition timestamps showed intervals of 22 s between the arterial and portal venous acquisitions and 6 min 06 s between the portal venous and delayed acquisitions. The TNC phase was part of the routine examination and was not added specifically for this report. The total dose–length product for the four-phase examination was 4246 mGy·cm, of which the TNC phase contributed 1060 mGy·cm (25%).

Postprocessing was performed on IntelliSpace Portal (Philips Healthcare). Low-keV monoenergetic images were reviewed only to improve enhancement conspicuity and to assist anatomical matching across phases; they were not used as the source of conventional attenuation values. Conventional polyenergetic images were used for Hounsfield-unit measurements, iodine-density maps displayed as overlays on the corresponding conventional images were used for IC, and standalone parametric maps were used for Zeff and EDW.

Two enhancement-defined intralesional compartments were identified: ROI-A, an early-enhancing component on arterial-phase review, and ROI-B, an initially hypoenhancing component that demonstrated delayed fill-in. Because the compartments were deliberately defined according to their contrasting postcontrast enhancement patterns, the subsequent quantitative analysis was intended to characterize predefined regional heterogeneity rather than independently discover divergent enhancement kinetics. To avoid phase-specific hotspot selection, each compartment was anatomically tracked through the TNC, arterial, venous, and delayed phases using linked axial review and stable anatomic landmarks rather than slice number alone. Circular ROIs were placed on three contiguous 3-mm reconstructions obtained at 1.5-mm intervals, avoiding lesion margins and, where present, visible vessels, calcification, visibly cystic or low-attenuation areas, hemorrhage, and partial-volume interfaces. ROI areas ranged from 33.8 to 40.7 mm^2^ and were adjusted when necessary to remain within the corresponding solid compartment. For each phase and compartment, the arithmetic mean of the three slice measurements was calculated to reduce dependence on a single axial level; this targeted approach was used as a pragmatic alternative to whole-lesion volumetric segmentation. The individual slice-level measurements are provided in Supplementary Table S1. No vascular normalization was applied because the analysis was intrapatient, intralesional, and focused on absolute physical values and temporal behavior. All measurements were performed by one reader (L.M.G.V.). Neither interobserver nor intraobserver reproducibility was assessed.

### 2.3. Spectral CT Findings and Clinical Interpretation

Conventional CT demonstrated a well-circumscribed solid right adnexal mass with spatially heterogeneous enhancement. Low-keV cine review emphasized early patchy enhancement and progressive delayed filling of initially hypoenhancing regions. The three-slice arithmetic means are summarized in Table 1, and the individual slice-level measurements are reported in Supplementary Table S1. Representative conventional and iodine-density images illustrating this enhancement pattern are shown in Figure 1.

Baseline TNC measurements. On TNC images, ROI-A and ROI-B showed closely similar attenuation (36.4 vs. 38.5 HU), Zeff (7.39 vs. 7.37), and EDW (103.8% vs. 103.9%). The low nonzero TNC iodine-map values (0.20 and 0.17 mg/mL) were treated as residual algorithmic signal rather than true iodine uptake. These findings demonstrated closely similar baseline spectral measurements in the two sampled solid compartments; they did not establish a specific tissue class, microscopic uniformity, or histologic subtype. Representative Zeff maps are shown in Figure 2.

Early-enhancing compartment (ROI-A). ROI-A showed intense arterial enhancement, with 102.2 HU, IC 2.95 mg/mL, and Zeff 8.78. These values decreased in the venous phase to 90.0 HU, IC 2.58 mg/mL, and Zeff 8.62, and decreased further in the delayed phase to 71.3 HU, IC 1.54 mg/mL, and Zeff 8.16. EDW showed lower-amplitude changes across the enhanced phases (105.2%, 104.8%, and 104.7% in the arterial, venous, and delayed phases, respectively).

Delayed-enhancing compartment (ROI-B). ROI-B showed low arterial and venous enhancement: arterial 50.9 HU, IC 0.67 mg/mL, and Zeff 7.69; venous 49.5 HU, IC 0.63 mg/mL, and Zeff 7.66. In the delayed phase, this compartment demonstrated marked fill-in, reaching 83.8 HU, IC 1.74 mg/mL, and Zeff 8.25. Its delayed mean values were slightly higher than those of ROI-A. The same late ordering was observed at all three sampled levels, including the two outer reconstructions with non-overlapping nominal slice volumes. Because the absolute delayed differences in IC and Zeff were small, this ordering is presented descriptively rather than as evidence of a validated quantitative distinction. EDW showed changes in the same general direction as enhancement but with lower amplitude (104.3%, 104.4%, and 105.1% in the arterial, venous, and delayed phases, respectively).

At the time of clinical reporting, the well-circumscribed morphology, solid stromal appearance, moderate heterogeneous enhancement, and absence of overt invasive features favored a benign stromal tumor, with fibroma/fibrothecoma considered the leading differential diagnosis. The detailed paired three-slice analysis was performed retrospectively for this report and did not alter the surgical decision prompted by the O-RADS 4 classification.

### 2.4. Surgical and Histopathological Correlation

The patient underwent right oophorectomy and uterine myomectomy. Operative findings described a discrete right ovarian mass measuring approximately 6 cm, excised together with the right ovary and anatomically separate from multiple uterine leiomyomas. On gross pathologic examination, the right ovarian specimen measured 5.5 × 3.5 × 2.6 cm, and the resected uterine leiomyoma measured 7 × 6 × 4.7 cm. The ultrasound, CT, operative, and gross pathologic measurements were obtained under different conditions and are therefore not directly interchangeable; their maximum dimensions were nevertheless broadly concordant. No pedicle or continuity between the ovarian mass and the uterus was described. Synchronous uterine leiomyomas coexist in many reported primary ovarian leiomyomas and do not preclude an ovarian origin when the ovarian mass is discrete and there is no evidence of direct uterine extension [22,23]. The postoperative course was uneventful.

Initial histopathological examination with hematoxylin and eosin staining provisionally suggested ovarian fibroma. Gross pathologic examination documented a 0.5-cm cystic-appearing cavity within the otherwise solid ovarian specimen; no corresponding discrete cystic component was identifiable on CT or transvaginal ultrasound. Immunohistochemistry demonstrated positivity for desmin, smooth muscle actin (SMA), and h-caldesmon, with negative beta-catenin and a reported Ki-67 proliferation index of 1%. The final pathologic diagnosis was ovarian leiomyoma (Figure 3). The myogenic immunophenotype, including h-caldesmon positivity, supported smooth-muscle differentiation consistent with the diagnosis [24,25].

## 3. Discussion

The retrospective analysis quantified the temporal behavior of two enhancement-defined intralesional compartments that were anatomically tracked across phases. ROI-A showed an arterial peak followed by progressive decline, whereas ROI-B showed low early enhancement with marked delayed fill-in. The slight delayed reversal, in which ROI-B exceeded ROI-A, is retained as a secondary descriptive observation. Although the compartments were selected because of their differing postcontrast enhancement patterns, their closely similar TNC measurements were not a selection criterion and were observed only after the regions had been defined.

The behavior of Zeff broadly paralleled regional iodine distribution. On TNC images, the two compartments had nearly identical Zeff values (7.39 and 7.37). After contrast administration, their Zeff values diverged markedly in the arterial phase (8.78 vs. 7.69), remained separated in the venous phase (8.62 vs. 7.66), and became slightly higher in ROI-B on delayed images (8.25 vs. 8.16), in parallel with the delayed iodine measurements. This phase- and region-dependent behavior is physically expected because high-atomic-number iodine contributes disproportionately to spectral attenuation [10,26]. Consequently, contrast-enhanced Zeff should be interpreted as a composite of underlying tissue and iodine present at the time of acquisition, rather than as an intrinsic tissue-composition measurement. A TNC acquisition provides the most direct iodine-free reference for Zeff, but the present findings do not support transferring any single Zeff threshold across platforms, phases, tumor types, or patient populations.

EDW showed a lower-amplitude phase-related response. TNC EDW was similar in the two compartments (103.8% and 103.9%), and the phase-ROI means ranged from 103.8% to 105.2%. The changes generally followed the direction of contrast enhancement but did not reproduce the magnitude of the regional differences observed in IC and Zeff. In the present case, EDW did not demonstrate diagnostic discrimination or histologic specificity, and its incremental contribution to adnexal mass characterization remains uncertain. Although electron density has shown potential value for tissue characterization in other organ systems [15,16,17,18,19,20,27], such findings cannot be extrapolated directly to ovarian leiomyoma.

Defining each compartment once and anatomically tracking it across phases allows the sequential values to represent the same targeted intralesional region over time; the approach remains targeted compartment sampling rather than a whole-lesion biomarker.

Importantly, spectral CT did not establish the histologic diagnosis in this patient and did not alter clinical management. Surgical management was driven by the O-RADS 4 assessment and the inability to exclude malignancy, whereas the detailed paired-compartment spectral analysis was performed retrospectively. Its contribution in this case was therefore descriptive characterization of intralesional enhancement heterogeneity rather than prospective diagnostic classification or management guidance.

Primary ovarian leiomyoma is rare and is seldom diagnosed preoperatively. Surgical series and case reports emphasize frequent confusion with ovarian fibroma, fibrothecoma, uterine leiomyoma, and malignant ovarian neoplasms [7,22,23,28,29,30]. This case adds quantitative dual-layer spectral CT characterization to a literature dominated by qualitative ultrasound, conventional CT, and MRI descriptions. Specifically, it contributes a paired multiphasic three-parameter dataset (IC, Zeff, and EDW) with slice-level measurements provided in Supplementary Table S1; an anatomically matched paired-compartment analysis across an already acquired multiphasic dual-layer spectral CT examination; and the observation that the two postcontrast-defined compartments had closely similar baseline TNC spectral measurements despite their intentionally selected contrasting enhancement patterns. Existing ovarian dual-energy CT studies have primarily evaluated cohort-level differentiation of benign, borderline, and malignant ovarian tumors [5,6,31]; these findings are phase-, population-, and platform-specific and should not be applied as single-case cutoffs to a rare mesenchymal tumor. These observations are descriptive and do not establish a spectral signature of ovarian leiomyoma or a generalizable diagnostic threshold.

MRI remains the preferred problem-solving modality for indeterminate adnexal masses [3]. In this patient, MRI was recommended but was not performed, precluding comparison with established T2-weighted and dynamic contrast-enhancement characteristics of ovarian stromal tumors [32]. The present dual-layer spectral CT findings should therefore not be interpreted as a substitute for MRI. Rather, they represent retrospective quantitative characterization of multiphasic dual-layer spectral CT data that had already been clinically acquired.

This report has several limitations. It describes a single case and cannot establish diagnostic thresholds or generalizable enhancement patterns. Measurements were performed by one reader, and neither interobserver nor intraobserver reproducibility was assessed. The two enhancement-defined compartments do not represent the entire lesion, and the three-slice mean is not equivalent to volumetric segmentation. The three-slice approach was intended to reduce dependence on a single axial level within each predefined compartment rather than to characterize whole-lesion heterogeneity; whole-lesion volumetric analysis would provide a more comprehensive lesion-level assessment in future studies. Because ROI-A and ROI-B were intentionally selected on the basis of their contrasting postcontrast enhancement patterns, the analysis quantified rather than independently discovered their divergent kinetics. Similarity of their TNC attenuation, Zeff, and EDW values was not a selection criterion and was observed only after the compartments had been defined. The three measurements represented contiguous spatial sampling rather than independent repeated acquisitions. Adjacent 3-mm reconstructions obtained at 1.5-mm intervals overlapped by 50%, although the two outer reconstructions had non-overlapping nominal slice volumes. Averaging reduced dependence on selection of a single axial level; between-slice variation should not be interpreted as measurement repeatability, statistical uncertainty, or instrument precision.

Although anatomical matching was performed carefully, small interphase differences in position and ROI contour cannot be eliminated. The TNC iodine values fell below 0.5 mg/mL, the concentration threshold below which iodine-quantification error on this platform increased markedly [33], supporting their interpretation as residual algorithmic signal rather than biologic iodine uptake. Contrast-phase values ranged from 0.63 to 2.95 mg/mL and therefore fell within the ≥0.5 mg/mL range for which Li et al. reported a mean absolute percentage error of approximately 13.7% [33]. Therefore, the divergent trajectories are more informative than the small delayed interregional differences in IC and Zeff, which are reported descriptively. Vascular normalization was intentionally omitted in this single-patient intralesional analysis, limiting direct comparison with normalized cohort studies. No direct spatial co-registration between imaging ROIs and histologic sections was available; therefore, the different enhancement trajectories cannot be attributed to specific microscopic features such as vascular density, fibrosis, or smooth-muscle content, and their histologic substrate remains undetermined. Because the small gross cystic-appearing cavity was not identifiable on imaging and no direct spatial co-registration between the imaging ROIs and the pathologic specimen was available, its relationship to ROI-B cannot be established and it cannot be used to explain the delayed fill-in pattern. MRI correlation was unavailable, limiting multimodality radiologic–pathologic comparison and preventing direct assessment of the lesion’s T2-weighted and dynamic contrast-enhancement characteristics. Exact patient-specific injection-to-acquisition delays were unavailable; nominal protocol timing and DICOM-confirmed interphase intervals are reported in Section 2.2. The approach is platform- and software-specific. Finally, although the operative findings described a discrete ovarian mass separate from the uterus, surrounding normal ovarian tissue was not specifically documented histologically.

The four-phase examination carried a total dose–length product of 4246 mGy·cm, representing a substantial radiation exposure. The examination was acquired clinically at a tertiary oncologic referral center using the institutional standard multiphasic abdominopelvic protocol for further characterization of the indeterminate adnexal mass and was not modified or extended for this retrospective analysis. All spectral results were generated retrospectively from the same acquisitions without additional scanning. These findings should not be interpreted as supporting multiphasic protocols or additional phases solely for spectral characterization; future applications should rely on clinically justified and dose-optimized acquisition strategies.

## 4. Conclusions

In this histologically confirmed ovarian leiomyoma, two enhancement-defined compartments showed closely similar TNC spectral measurements, while postcontrast IC and Zeff quantified their differing enhancement trajectories; EDW showed lower-amplitude changes without histologic specificity. The spectral findings did not establish the histologic diagnosis or alter management. When multiphasic dual-layer spectral CT has already been clinically acquired, regional analysis may provide descriptive information on intralesional enhancement heterogeneity, but these single-case findings do not establish diagnostic thresholds or a specific spectral signature, and histopathology with immunohistochemistry remains necessary for definitive diagnosis.

## Figures and Tables

**Figure 1:**
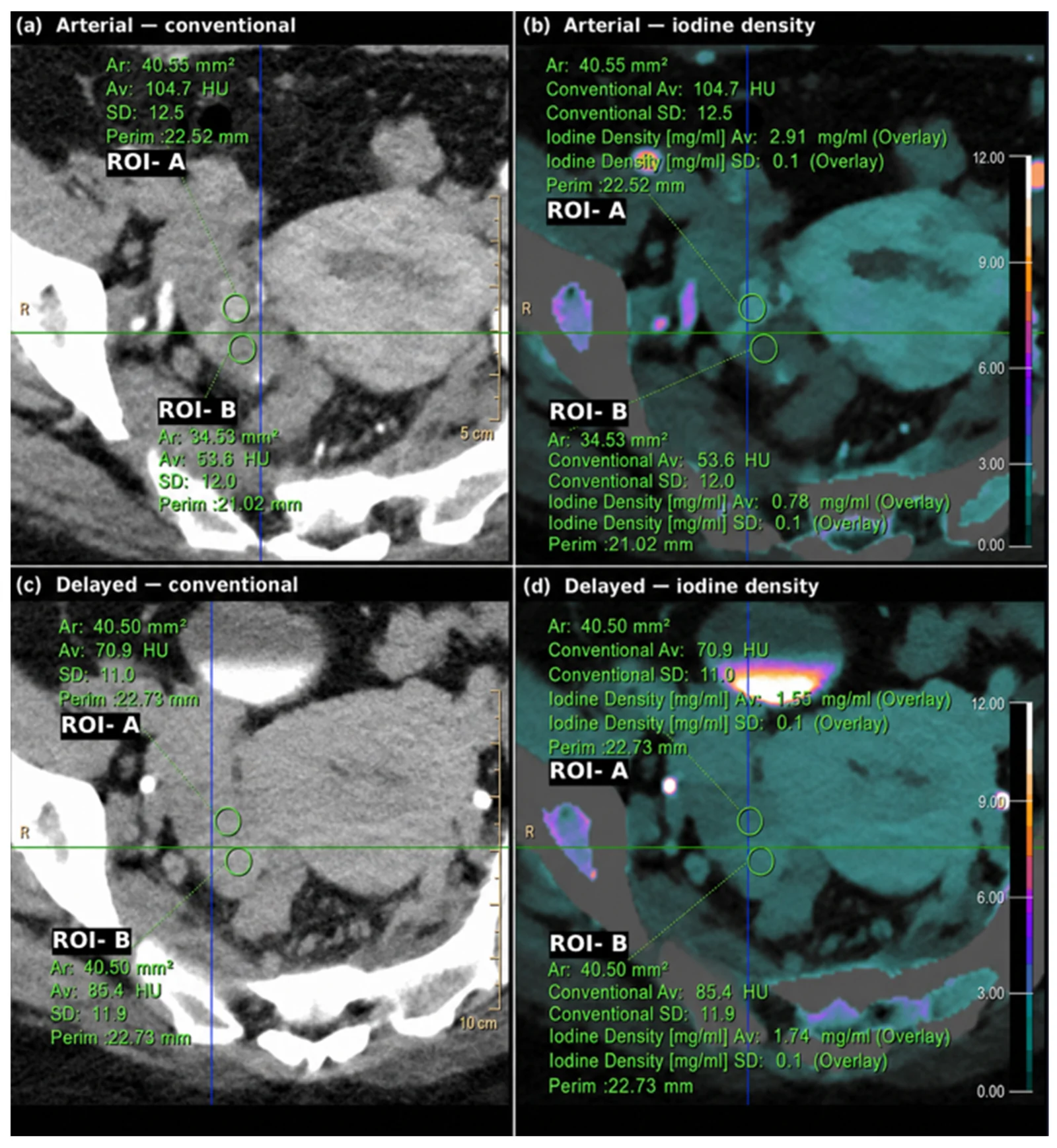
Conventional CT and iodine-density overlays at the representative central slice for the arterial and delayed phases. (**a**) Arterial conventional CT. (**b**) Arterial iodine-density overlay. (**c**) Delayed conventional CT. (**d**) Delayed iodine-density overlay. ROI-A and ROI-B are explicitly labeled in all panels. ROI-A (early-enhancing compartment) demonstrates intense arterial iodine uptake (**a**,**b**) followed by decline; ROI-B (initially hypoenhancing compartment) remains low in the arterial phase and demonstrates delayed fill-in, with its three-slice mean delayed attenuation and iodine concentration slightly exceeding those of ROI-A (**c**,**d**). The venous phase, which confirms persistence of the arterial separation, is shown in Video S1. Table 1 reports three-slice arithmetic means; the displayed images represent the central slice.

**Figure 2:**
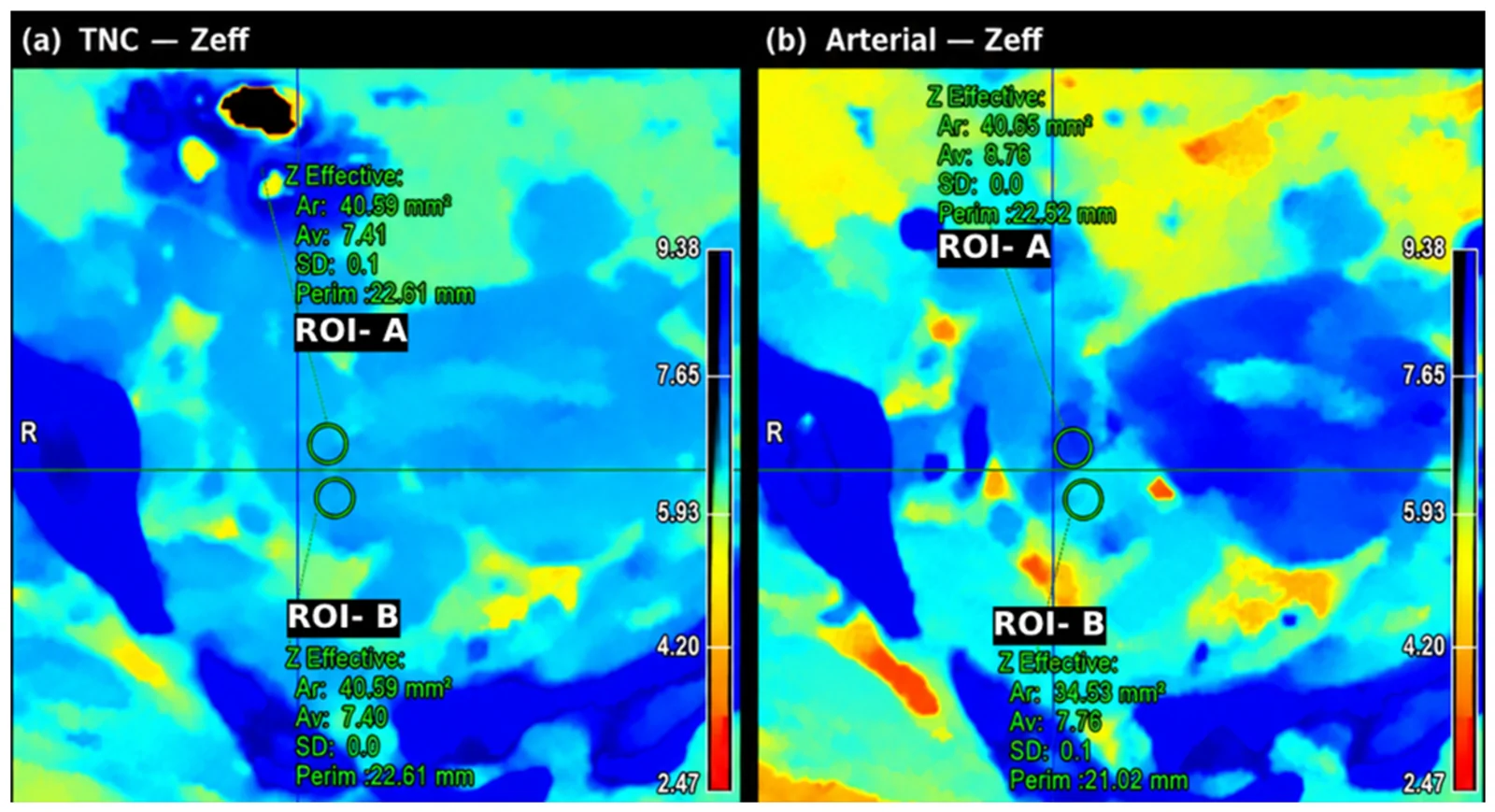
Standalone effective atomic number (Zeff) maps at the representative central slice for the TNC and arterial phases. (**a**) TNC Zeff. (**b**) Arterial Zeff. ROI-A and ROI-B show closely similar Zeff values on TNC imaging, whereas their arterial-phase Zeff values diverge in parallel with regional contrast distribution. Table 1 reports the three-slice arithmetic means; the displayed images represent the central slice.

**Figure 3:**
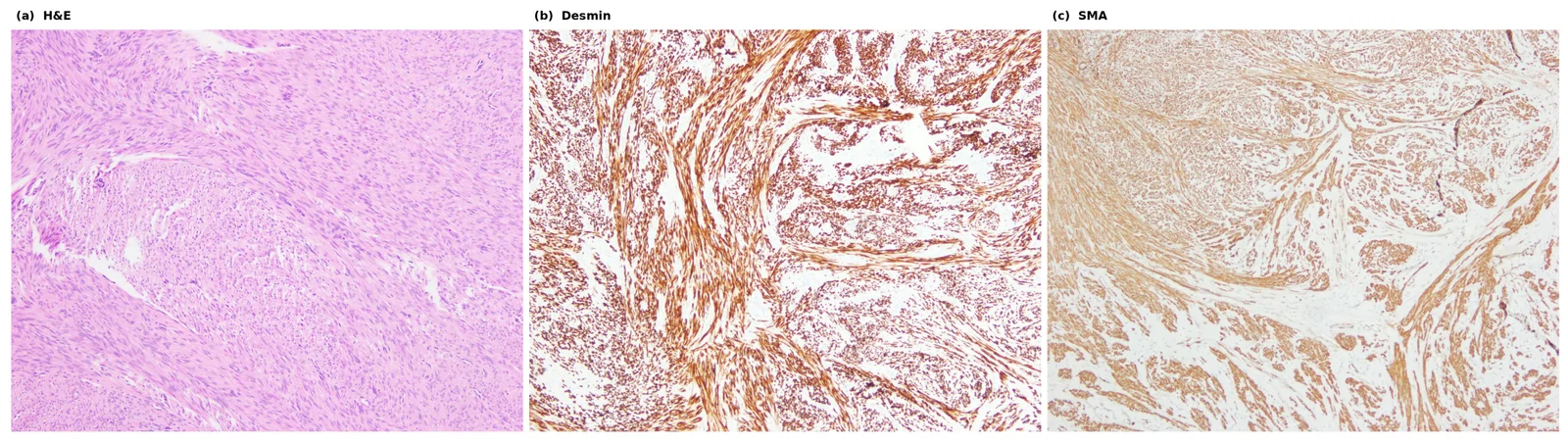
Histopathology and immunohistochemistry. (**a**) Hematoxylin and eosin staining showing spindle-cell morphology. (**b**) Desmin positivity. (**c**) Smooth muscle actin positivity. Immunohistochemistry also demonstrated h-caldesmon positivity, negative beta-catenin, and a reported Ki-67 proliferation index of 1%. The final pathologic diagnosis was ovarian leiomyoma. Original magnification: (**a**–**c**) ×100.

**Table 1:** Three-slice multiparametric spectral CT measurements in the early-enhancing compartment (ROI-A) and delayed-enhancing compartment (ROI-B). Values are arithmetic means of three anatomically matched slice measurements.

Phase	Region	Conventional HU	IC (mg/mL)	Zeff	EDW (%)
TNC	ROI-A (early)	36.4	0.20 *	7.39	103.8
TNC	ROI-B (delayed)	38.5	0.17 *	7.37	103.9
Arterial	ROI-A (early)	102.2	2.95	8.78	105.2
Arterial	ROI-B (delayed)	50.9	0.67	7.69	104.3
Venous	ROI-A (early)	90.0	2.58	8.62	104.8
Venous	ROI-B (delayed)	49.5	0.63	7.66	104.4
Delayed	ROI-A (early)	71.3	1.54	8.16	104.7
Delayed	ROI-B (delayed)	83.8	1.74	8.25	105.1

* TNC iodine-density values were below 0.5 mg/mL and represent low-level residual algorithmic signal rather than biologic iodine uptake. Adjacent 3-mm reconstructions obtained at 1.5-mm intervals overlapped by 50%; the two outer reconstructions had non-overlapping nominal slice volumes. Individual slice-level values are provided in Supplementary Table S1. Between-slice variation represents local spatial variability and should not be interpreted as measurement repeatability or statistical uncertainty. ROI areas ranged from 33.8 to 40.7 mm^2^. Conventional HU were measured on conventional images; IC on iodine-density overlays; Zeff and EDW on standalone maps. No vascular normalization was used. TNC, true noncontrast; IC, iodine concentration; Zeff, effective atomic number; EDW, electron density relative to water.

## Data Availability

The data supporting the findings of this study are available from the corresponding author upon reasonable request. The de-identified slice-level quantitative measurements are provided in Supplementary Table S1. The source spectral CT datasets are not publicly available because of patient privacy regulations.

## Supplementary Materials

The following supporting information can be downloaded at https://www.mdpi.com/article/10.3390/diagnostics16203420/s1: Table S1: Individual slice-level conventional and spectral measurements for ROI-A and ROI-B across the TNC, arterial, venous, and delayed phases. Video S1: Multiphasic low-keV monoenergetic cine including TNC, arterial, venous, and delayed phases, demonstrating patchy early enhancement and progressive delayed fill-in.

## Abbreviations

The following abbreviations are used in this manuscript:

CT	computed tomography
CTDIvol	volume computed tomography dose index
EDW	electron density relative to water
HU	Hounsfield unit
IC	iodine concentration
MRI	magnetic resonance imaging
O-RADS	Ovarian–Adnexal Reporting and Data System
ROI	region of interest
SMA	smooth muscle actin
TNC	true noncontrast
Zeff	effective atomic number
